# Employment status and income as potential mediators of educational inequalities in population mental health

**DOI:** 10.1093/eurpub/ckw126

**Published:** 2016-09-04

**Authors:** Srinivasa Vittal Katikireddi, Claire L. Niedzwiedz, Frank Popham

**Affiliations:** 1 MRC/CSO Social and Public Health Sciences Unit, University of Glasgow, Top floor, 200 Renfield Street, Glasgow, G2 3QB, UK; 2 Centre for Research on Environment, Society and Health, University of Edinburgh, Drummond Street, Edinburgh, EH8 9XP, UK

## Abstract

We assessed whether educational inequalities in mental health may be mediated by employment status and household income. Poor mental health was assessed using General Health Questionnaire ‘caseness’ in working age adult participants (*N* = 48 654) of the Health Survey for England (2001–10). Relative indices of inequality by education level were calculated. Substantial inequalities were apparent, with adjustment for employment status and household income markedly reducing their magnitude. Educational inequalities in mental health were attenuated by employment status. Policy responses to economic recession (such as active labour market interventions) might reduce mental health inequalities but longitudinal research is needed to exclude reverse causation.

## Introduction

Mental health is socially patterned—the more disadvantaged in society consistently experience poorer outcomes.^1^ Social inequalities in common mental disorders, including anxiety and depression arise, at least in part, as a result of adverse socio-economic circumstances. Addressing the wider determinants of health, including employment status and working conditions, is repeatedly prioritised in policy reports.^2^ Recent changes in the global economy are resulting in job loss across Europe, raising concerns that health inequalities may widen.

In addition to being a dimension of social stratification in itself, employment status is an important determinant of mental health and vice-versa.^3^ High-quality work not only delivers an income to buy necessities for living, it also provides a sense of purpose and order to life, whereas poor mental health may lead to labour market exclusion. Major differences exist between social groups in employment status and its consequences for ill-health^4^; thus differences in employment status and income could contribute to health inequalities. Therefore, both employment status and income may be important potentially modifiable factors along the pathway between education level (a measure of early adulthood socioeconomic position) and later life. We therefore investigated the extent to which educational inequalities in mental health may be mediated by employment status and income among working age adults in England.

## Methods

Annual data were taken from the 2001 to 2010 Health Surveys for England, nationally representative cross-sectional surveys of the community dwelling population. These data were pooled to provide adequate statistical power. Survey methodology is described in detail elsewhere^5^; household response rates varied from 74% in 2001 to 64% in 2008 and ethical approval was obtained by each survey team.

The study population was restricted to working age participants (25–64 years inclusive). Similar results were obtained when limiting the sample to 25–59 years. Participants missing any data for age, sex, highest education level, employment status and the outcome were excluded (19.7% of participants, with those missing data on covariates tending to have slightly worse mental health).

A conceptual model informing the analysis is presented as a Supplementary Figure. Socio-economic position was assessed using highest education level, a measure of early adulthood socioeconomic position. We also conducted a sensitivity analysis using area-level deprivation (assessed using the index of multiple deprivation). Two mediating factors were investigated: employment status based on participants’ activity in the week prior to the survey interview and equivalized household income. Further details of the study population and coding of variables have been published elsewhere.^6^

The outcome measure was poor mental health, defined by a score of four or more on the General Health Questionnaire (GHQ-12): GHQ ‘caseness’. GHQ-12 is a screening tool for minor psychiatric morbidity, particularly anxiety and distress, which has been validated for epidemiological studies.^7^ Similar results were obtained with continuous GHQ-12 scores.

To quantify socio-economic inequalities, relative indices of inequality (RIIs) were calculated. Educational level was ranked from the least to most advantage (with the mid-point of their range in the cumulative distribution used for each category). These were standardized to produce an index ranging from zero to one. Year-specific ranks were calculated to allow for secular changes in the distribution of socio-economic position measures, but sensitivity analyses using rank measures calculated from the pooled data produced similar findings. We also investigated whether associations varied over time, given the recession during the time period analysed. The RII was calculated by running Poisson regression models (to provide readily interpretable results that allow comparison across models^8^), adjusted for age. On the basis of theory and confirmed by a statistically significant interaction term, models were stratified by gender. Coefficients are expressed as prevalence risk ratios (PRRs) which can be interpreted as the relative risk for GHQ caseness for the theoretically least advantaged compared with the most advantaged person. Potential mediating factors, employment status and income, were added in turn. Analyses were carried out using Stata v13.1. Clustering was accounted for at the area-level and weights used when available (from 2003 onwards).

## Results

Data from 21 826 male and 26 828 female participants were included in the analysis. Pooled across the time period, the prevalence of GHQ caseness amongst males was 12.2% (95% CI: 11.7–12.7%) and 16.0% (95% CI: 15.6–16.5%) in females. Poor mental health varied by employment status; the employed had lower GHQ caseness (10.7%) than those who were out of work due to ill health (50.1%) or unemployment (28.5%).

Poor mental health was socially patterned by education level (Table 1). Although the prevalence of poor mental health was higher among women, socio-economic inequalities were larger among men. The PRR for highest education level was 1.86 (95% CI: 1.62–2.14) for men and 1.48 (95% CI: 1.32–1.66) for women.

**Table 1:** RII for GHQ caseness in the Health Survey for England (2001–10), with adjustment for age, employment status and household income

Covariates	Age		Age + employment status		Age + income		Age + employment status + income	
	PRR	95% CI	PRR	95% CI	PRR	95% CI	PRR	95% CI
**Men**								
RII by education	1.86	1.62–2.14	0.96	0.83–1.11	1.06	0.91–1.23	0.87	0.75–1.01
**Age**								
25-34 (ref)								
35–44	1.05	0.94–1.17	1.03	0.93–1.15	1.07	0.96–1.19	1.03	0.93–1.15
45–54	1.09	0.98–1.22	1.00	0.89–1.11	1.14	1.02–1.28	1.01	0.9–1.12
55–64	0.98	0.87–1.10	0.77	0.68–0.87	1.00	0.89–1.12	0.79	0.70–0.89
Employed (ref)								
Unemployed			3.14	2.71–3.63			2.72	2.32–3.20
Not working due to ill health			5.88	5.42–6.39			5.16	4.66–5.72
Retired			1.27	1.03–1.55			1.2	0.97–1.47
Looking after home/family			2.48	1.97–3.12			2.18	1.72–2.75
In education			1.39	1.03–1.88			1.3	0.96–1.77
Highest income quintile 1 (ref)								
Quintile 2					1.06	0.94–1.20	1.07	0.94–1.21
Quintile3					1.24	1.10–1.41	1.12	0.99–1.27
Quintile 4					1.83	1.62–2.07	1.25	1.10–1.43
Lowest Quintile 5					3.13	2.79–3.51	1.33	1.16–1.53
**Women**								
RII by education	1.48	1.32–1.66	1.05	0.95–1.18	0.92	0.82–1.04	0.83	0.74–0.94
**Age**								
25–34 (ref)								
35–44	1.03	0.95–1.11	1.03	0.95–1.11	1.05	0.97–1.13	1.03	0.95–1.12
45–54	1.16	1.07–1.26	1.08	1.00–1.18	1.25	1.15–1.36	1.12	1.04–1.22
55–64	0.96	0.88–1.05	0.88	0.80–0.97	1.02	0.93–1.11	0.92	0.83–1.01
Employed (ref)								
Unemployed			2.45	2.05–2.93			2.02	1.68–2.43
Not working due to ill health			4.06	3.77–4.36			3.41	3.15–3.69
Retired			1.33	1.17–1.52			1.21	1.06–1.39
Looking after home/family			1.37	1.27–1.48			1.22	1.12–1.32
In education			1.6	1.29–1.98			1.44	1.16–1.78
Highest income quintile 1 (ref)								
Quintile 2					1.15	1.05–1.27	1.14	1.04–1.26
Quintile3					1.33	1.21–1.47	1.25	1.13–1.38
Quintile 4					1.63	1.48–1.80	1.4	1.27–1.54
Lowest Quintile 5					2.35	2.14–2.58	1.71	1.55–1.89

RII, Relative Index of Inequality; PRR, Prevalence Risk Ratio.

Both employment status and income were associated with education level (see Supplementary Tables S1a–c). Adjustment for either employment status or income entirely, or almost entirely, abolished educational inequalities in mental health (Table 1). There was a suggestion that attenuation varied by gender, with employment status accounting for more of the inequalities among men and income appeared to play a greater role in women. Models which included both employment status and income found independent associations for both, with the gradient in poor mental health reversed.

Sensitivity analysis based on area-based deprivation yielded similar results to our main analysis (see Supplementary Tables S2a and b). Exclusion of those not working due to ill health resulted in smaller educational inequalities but a similar pattern of results Supplementary Tables S3a and b. Last, we investigated whether findings differed over time since the global economic downturn occurred during the study period (Supplementary Tables S4a and b). Data were stratified into three time periods (2001–03, 2004–06 and 2008–10) and associations between employment status and mental health were slightly weaker during the period of recession, but there was substantial variability between pre-recession periods as well. Inequalities have increased slightly over time but there was little variation in attenuation.

## Discussion

Socioeconomic inequalities in poor mental health, as assessed by education level, were consistently observed in the working-age population of England. These inequalities were abolished after adjustment for either employment status or income—both important and potentially modifiable factors. Our findings therefore raise the possibility that employment status may act as an important mediator of socioeconomic inequalities in mental health.

To our knowledge, the contribution of differential patterning of worklessness has not previously been explored for mental health inequalities. However, differential social patterning of worklessness has been related to inequalities in poor self-rated health.^9^,^10^ The patterning of findings by gender, with employment status mediating a greater proportion of the inequalities in mental health in men, is noteworthy—especially, given the consistent finding that men’s health is more likely to be affected by recessions.^3^,^6^

This study has a number of strengths: the use of a rigorously conducted series of population representative surveys; a large sample size and the validated outcome measure. However, given the cross-sectional nature of the surveys, reverse causation is possible and longitudinal research is needed.

The association between employment status and mental health inequalities highlights the potential importance of changes in employment and welfare for health inequalities. If the observed association is causal, a key mechanism for addressing mental health inequalities may include active labour market policies to reduce worklessness.

## Supplementary data

Supplementary data are available at *EURPUB* online.

## References

[1] Allen J Balfour R Bell R Marmot M Social determinants of mental health. Int Rev Psychiatry 2014;26:392–407. 25137105 10.3109/09540261.2014.928270

[2] European Commission Health inequalities in the EU—Final report of a consortium. Consortium lead: Sir Michael Marmot. European Commission Directorate-General for Health and Consumers 2013.

[3] Bambra C Work, Worklessness, and the Political Economy of Health. Oxford: Oxford University Press, 2011. 10.1136/jech.2009.102103 21282147

[4] Minton JW Pickett KE Dorling D Health, employment, and economic change, 1973-2009: repeated cross sectional study. Br Med J 2012;344:e2316. 22573646 10.1136/bmj.e2316 PMC3348868

[5] Mindell J Biddulph JP Hirani V, Cohort Profile: The Health Survey for England. Int J Epidemiol 2012;41:1585–93. 22253315 10.1093/ije/dyr199

[6] Katikireddi SV Niedzwiedz CL Popham F Trends in population mental health before and after the 2008 recession: a repeat cross-sectional analysis of the 1991–2010 Health Surveys of England. BMJ Open 2012;2:e001790. 10.1136/bmjopen-2012-001790 PMC3488736 23075569

[7] Goldberg DP Gater R Sartorius N, The validity of two versions of the GHQ in the WHO study of mental illness in general health care. Psychol Med 1997;27:191–7. 9122299 10.1017/s0033291796004242

[8] Barros A Hirakata V Alternatives for logistic regression in cross-sectional studies: an empirical comparison of models that directly estimate the prevalence ratio. BMC Med Res Method 2003;3:21. 10.1186/1471-2288-3-21 PMC521200 14567763

[9] Bambra C Popham F Worklessness and regional differences in the social gradient in general health: Evidence from the 2001 English census. Health Place 2010;16:1014–21. 20638320 10.1016/j.healthplace.2010.06.006

[10] Popham F Bambra C Evidence from the 2001 English Census on the contribution of employment status to the social gradient in self-rated health. J Epidemiol Commun Health 2010;64:277–80. 10.1136/jech.2009.087452 19692722

